# Embryonic Hyperglycemia Disrupts Myocardial Growth, Morphological Development, and Cellular Organization: An In Vivo Experimental Study

**DOI:** 10.3390/life13030768

**Published:** 2023-03-13

**Authors:** Ricardo Jaime-Cruz, Concepción Sánchez-Gómez, Laura Villavicencio-Guzmán, Roberto Lazzarini-Lechuga, Carlos César Patiño-Morales, Mario García-Lorenzana, Tania Cristina Ramírez-Fuentes, Marcela Salazar-García

**Affiliations:** 1 Posgrado en Biología Experimental, Departamento de Ciencias de la Salud, Universidad Autónoma Metropolitana-Iztapalapa, Mexico City 09340, Mexico; 2 Research Laboratory of Developmental Biology and Experimental Teratogenesis, Children’s Hospital of México Federico Gomez, Mexico City 06720, Mexico; 3 Departamento de Ciencias de la Salud, Universidad Tecnológica de México—UNITEC México—Campus Sur, Mexico City 09810, Mexico; 4 Departamento de Biología de la Reproducción, División de Ciencias Biológicas y de la Salud, Universidad Autónoma Metropolitana-Iztapalapa, Mexico City 09340, Mexico; 5 Laboratorio de Biología Celular, Universidad Autónoma Metropolitana-Cuajimalpa, Mexico City 05348, Mexico; 6 Sección de Estudios de Posgrado e Investigación de Escuela Superior de Medicina, Instituto Politécnico Nacional, Mexico City 11340, Mexico; 7 Facultad de Medicina, Universidad Nacional Autónoma de México, Mexico City 04360, Mexico

**Keywords:** hyperglycemia, myocardial growth, cardiomyopathies, heart development

## Abstract

Hyperglycemia during gestation can disrupt fetal heart development and increase postnatal cardiovascular disease risk. It is therefore imperative to identify early biomarkers of hyperglycemia during gestation-induced fetal heart damage and elucidate the underlying molecular pathomechanisms. Clinical investigations of diabetic adults with heart dysfunction and transgenic mouse studies have revealed that overexpression or increased expression of TNNI3K, a heart-specific kinase that binds troponin cardiac I, may contribute to abnormal cardiac remodeling, ventricular hypertrophy, and heart failure. Optimal heart function also depends on the precise organization of contractile and excitable tissues conferred by intercellular occlusive, adherent, and communicating junctions. The current study evaluated changes in embryonic heart development and the expression levels of sarcomeric proteins (troponin I, desmin, and TNNI3K), junctional proteins, glucose transporter-1, and Ki-67 under fetal hyperglycemia. Stage 22HH *Gallus domesticus* embryos were randomly divided into two groups: a hyperglycemia (HG) group, in which individual embryos were injected with 30 mmol/L glucose solution every 24 h for 10 days, and a no-treatment (NT) control group, in which individual embryos were injected with physiological saline every 24 h for 10 days (stage 36HH). Embryonic blood glucose, height, and weight, as well as heart size, were measured periodically during treatment, followed by histopathological analysis and estimation of sarcomeric and junctional protein expression by western blotting and immunostaining. Hyperglycemic embryos demonstrated delayed heart maturation, with histopathological analysis revealing reduced left and right ventricular wall thickness (−39% and −35% vs. NT). Immunoexpression levels of TNNI3K and troponin 1 increased (by 37% and 39%, respectively), and desmin immunofluorescence reduced (by 23%). Embryo-fetal hyperglycemia may trigger an increase in the expression levels of TNNI3K and troponin I, as well as dysfunction of occlusive and adherent junctions, ultimately inducing abnormal cardiac remodeling.

## 1. Introduction

Diabetes mellitus (DM) is a chronic condition characterized by the dysregulation of physiological blood glucose levels, leading to periodic hyperglycemia. It is now considered a primary public health crisis owing to its rapidly growing prevalence in many regions of the world and the severity of DM-associated complications, especially cardiovascular diseases and neuropathies [1,2].

Pregnancy is associated with major physiological changes, including dysregulation of blood glucose [3,4]. According to the estimates of the International Diabetes Federation (IDF), 16.2% of women worldwide who delivered live children in 2017 had some form of hyperglycemia during pregnancy, of which an estimated 86.4% of cases were due to gestational DM (GDM), 6.2% to pregestational DM, and 7.4% to diabetes onset during pregnancy. In addition to high glucose levels, GDM is characterized by hyperinsulinemia and alterations in other hormones produced by the placenta. These changes can delay embryonic development and contribute to higher offspring morbidity and mortality [5,6].

Hyperglycemia during pregnancy can harm progeny in the short, medium, and long terms. In the short term, hyperglycemia is associated with various congenital defects, including heart malformations, which are a leading cause of intrauterine and perinatal death. Furthermore, maternal hyperglycemia and lifestyle during pregnancy can affect prenatal and postnatal development [2] and increase the risks of DM, obesity, and cardiovascular disease in both childhood and adulthood [4]. Despite the high incidence of hyperglycemia during gestation and its deleterious effects on offspring, the cellular and molecular mechanisms that lead to the development of these diseases have not been fully elucidated.

Normal embryonic heart development depends on proper sequential specifications of the mesoderm, splanchnic mesoderm, and cardiogenic mesoderm, followed by morphogenesis of the embryonic cardiac chambers that ultimately form the mature four-chambered heart [7,8,9]. Ventricular growth and development in mammals are highly dependent on the changes that occur in the cardiomyocyte population [10,11]. Failure in any morphogenetic process or gene expression program during cardiogenesis can lead to various morphological and functional defects.

Hyperglycemia is associated with multiple cardiovascular diseases, including cardiomyopathies, that result in insufficient ventricular strength for efficient circulation [11,12]. Cardiomyopathies may result from gross malformation or finer abnormalities in the microstructural organization of contractile elements and cellular pathways that transmit electrical excitation with the optimal spatiotemporal pattern for maximal force generation. In such disorders, the ventricular myocardium may become dilated, and the walls thinner, resulting in progressive heart failure and potential sudden death [13,14].

While the statistical links between hyperglycemia, diabetes, and myocardiopathies are well established among adults and children descended from diabetic women, the pathogenic processes leading to morphological and functional abnormalities in offspring are unclear.

The domestic chicken (*Gallus domesticus*) embryo is a potentially valuable model for studying the pathogenesis of fetal cardiovascular abnormalities in a hyperglycemic environment because it allows for detailed morphological analyses throughout gestation [15], can be easily manipulated to mimic some of the microenvironmental conditions of human development independent of maternal status, including hyperglycemia [16], and is inexpensive enough to generate large sample numbers during a single incubation. The chicken embryo has been used as a model to investigate the effects of hypoxia on fetal development [17].

The aim of this study was to analyze the development of the embryonic ventricular myocardium under induced hyperglycemia, including morphological parameters and possible influences on sarcomeric proteins, cell junction proteins, cell proliferation, and cellular glucose transport. Collectively, these findings implicate dysregulation as a critical event in the disruption of cardiac structure and function under hyperglycemia and point out multiple targets for prenatal diagnosis.

## 2. Materials and Methods

### 2.1. Embryos

Fertilized Bovans chicken eggs were obtained from a local poultry farm (ALPES, Puebla, Mexico) and incubated at 37.8 °C under 60% relative humidity until they reached stage 22HH (3–3.5 days). The eggshells were then disinfected with 70% alcohol and windowed (2 cm^2^) to stage the embryos, followed by exposure of the heart via dissection of the allantoidal and pericardial membranes (total n = 87 embryos). The eggs were then randomly divided into two groups: a hyperglycemic group (HG, n = 44) and a normoglycemic group with no treatment (NT, n = 43). To induce hyperglycemia, the HG embryos were treated according to the methodology of Zhang (2016) with modifications. In each HG group egg, a 450 µL volume of 30 mmol/L glucose in saline (NaCl 0.9%) was injected daily through the shell window using a 1 mL syringe to a maximum of 10 days (36HH stage), while the NT group eggs received daily injection of 450 µL saline. To verify the induction of hyperglycemia, the HG and NT embryos were obtained every 24 h starting on day 4 of incubation, separated from the yolk, and decapitated. A small blood sample was then taken to record blood glucose using test strips and a glucometer (Freestyle, Abbott Laboratories, Chicago, IL, USA). The animal use protocols and study procedures strictly conformed to Mexican Official Guidelines (NOM-062-ZOO-1999) and were approved by the research, ethics, and biosafety committees of the Children’s Hospital of Mexico Federico Gomez (HIM/2018/057. SSA-156).

### 2.2. Weight and Body Development

Embryonic development was assessed using an epifluorescence stereoscopic microscope (Zeiss, Jena, Germany) with Axiovision LE software. Once the embryos were photographed, they were weighed on an analytical balance and the heart was removed for imaging using the above-mentioned stereoscopic epifluorescence microscope (HG, n = 23; NT, n = 23).

### 2.3. Histology

Following examination of gross morphology, hearts from stage 36HH embryos (HG, n = 8; NT, n = 9) were fixed in neutral formalin, dehydrated through a graded alcohol series (70–100%), rinsed in xylene (Sigma-Aldrich, Burlington, MA, USA), embedded in Paraplast wax (Tissue-Tek, CA, USA), cut into 6 µm cross-sections at the level of the papillary muscles, and stained with hematoxylin and eosin (HE; Hycel, Jalisco, Mexico). The total diameter of each ventricular wall and the myocyte count in the three regions of interest were measured using a Digital Aperio Scanner (Leica, Wetzlar, Germany) and ImageScope software.

### 2.4. Scanning Electron Microscopy

Stage 36HH hearts (HG, n = 6; NT, n = 7) fixed and photographed as described above were sectioned in transversal and longitudinal planes, dehydrated, desiccated under liquid CO_2_ in a critical-point drying apparatus (Samdri 789A, Tousimins Research Co., Rockville, MD, USA), and sputter-coated with 350 nm gold in a Denton Vacuum Desk 1A apparatus (Denton, Cherry Hill Industrial Centre, Cherry Hill, NJ, USA). Electron micrographs were acquired using a JSM 5300 Scanning Electron Microscope (JEOL, Tokyo, Japan) at 15 kV and various magnifications, as indicated in the figures. These electron micrographs were used to illustrate the organization of the ventricular trabeculae and to visualize the compaction process of the ventricular walls.

### 2.5. Immunostaining and Detection by Confocal Microscopy

Protein expression levels were estimated by immunohistochemistry using primary antibodies (1:200 dilution) against TNNI3K, troponin I, desmin, N-cadherin,β-catenin, claudin-1, ZO-1 (all from Santa Cruz Biotechnology, Dallas, TX, USA), Cx43, and GLUT1 (Novus, Littleton, CO, USA). The samples were incubated with primary antibodies overnight at 4 °C before being treated with fluorescence-tagged anti-rabbit secondary antibodies (Santa Cruz, Dallas, TX, USA; SC2359) for 4 h at room temperature. The cell nuclei were counterstained with RedDot (1:150, Biotium, Freemont, CA, USA) diluted 1:150. Finally, the samples were mounted and viewed under a confocal microscope (Carl Zeiss, Oberkochen, Germany). Sections were obtained at the level of the papillary muscles in the 3 areas of interest and imaged at 10×, 40×, and 60× magnification using ZEN 2010 software (Carl Zeiss, Germany). The average optical density from each protein–antibody complex is reported as the mean (±standard deviation, SD) relative to the NT group (set to 100%) (n = 9 sections from HG and n = 10 sections from NT group hearts).

### 2.6. Western Blotting

Ventricular tissues from 36HH stage hearts (n = 6 each from HG and NT groups) were placed in individual Eppendorf tubes with Tris-HCl lysis solution plus protease inhibitor, homogenized, and centrifuged. Total protein concentration in the supernatant was measured by the absorbance at 280 nm using a nanodrop spectrophotometer (Fisher Scientific, Waltham, MA, USA). Proteins were separated on 12% polyacrylamide gels and transferred to PVDF membranes (Bio-rad) using a Trans-Blot apparatus (Fisher Scientific). The Precision Plus Protein Dual Color Standards (Bio-Rad, Hercules, CA, USA) were used as molecular weight markers. Membranes were labeled with primary antibodies against TNNI3K, troponin I, desmin, Cx43, N-cadherin, β-catenin, claudin-1, ZO-1, GLUT1 (suppliers provided in the previous section), and actin as the gel-loading control (all from Santa Cruz Biotechnology, Dallas, TX, USA). Protein bands were quantified using chemiluminescence and expressed as mean ± SD relative to corresponding bands from the NT group (set to 100%) (HG, n = 9 gels; NT, n = 10 gels).

### 2.7. Statistical Analysis

Weight, height, blood glucose, morphometric parameters, and immunolabeling results expression measurements were first assessed for normality using the Shapiro–Wilk test and expressed as mean ± SD. Morphometric measurements were compared between groups using a two-tailed Student’s *t*-test. A *p* < 0.05 was considered significant for all tests.

Morphometric measurements were compared between groups using one-way ANOVA and Student’s *t*-test using independent samples. A *p* < 0.05 (two-tailed) was considered significant for all tests.

## 3. Results

### 3.1. Induction of Hyperglycemia

**Figure 1:**
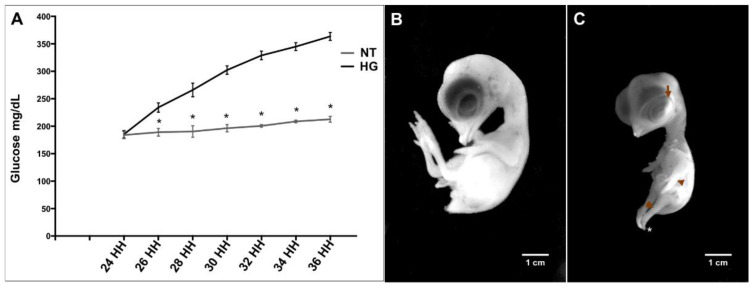
Delayed maturation of hyperglycemic *Gallus* embryos compared to normoglycemic embryos. (**A**) Changes in blood glucose levels after daily injections of 30 mmol/L glucose saline in the hyperglycemic (HG) group and daily injections of glucose-free saline in the control NT group. (**B**) *Gallus* embryo incubated for 10 days under normoglycemic conditions reaching stage 36HH (n = 32 independently treated embryos in each group). (**C**) *Gallus* embryos incubated under hyperglycemia for 10 days showing delayed maturation (typical example from HG-treated embryos). Arrowhead, delayed limb maturation; arrow, delayed eyelid maturation; star, defects in leg phalanges. (*) *p* < 0.005, (scale bar = 1 cm).

In both HG and NT groups, embryonic development was allowed to continue for 10 days, or to stage 36HH under normal conditions, with daily injections of high glucose and normal saline, respectively. In the HG group embryos, blood glucose levels increased progressively (*p* = 0.0029), with the mean value surpassing 350 mmol/dL by day 10, while the NT embryos showed no significant changes (mean 198 mmol/dL) (Figure 1A).

### 3.2. Weight and Body Development

Embryos from the HG group exhibited significantly reduced mean weight and size, and a general delay in maturation, compared to NT group embryos. Most NT embryos reached the expected 36HH stage by day 10, while the HG embryos reached only the 33HH stage. In addition, the HG embryos exhibited delayed limb and eyelid maturation, as well as defects in the phalanges of the legs (Figure 1B,C).

**Scheme 1:**
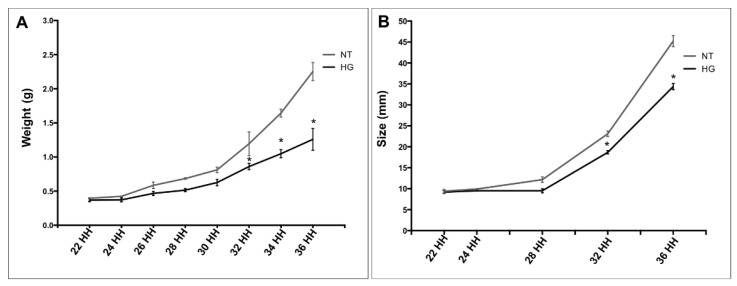
Hyperglycemia stunts embryonic growth. (**A**) Mean body weight of the HG embryos was 34% lower than that of the NT embryos on day 10. (**B**) Mean body size of the HG embryos was 14% shorter than that of the NT embryos on day 10. Graphs represent mean ± SD of n = 23 HG and n = 25 NT embryos. (*) *p* < 0.05.

Body weight (Scheme 1A) and body length (Scheme 1B) increased exponentially in both groups during embryonic development, but the mean body weight of day-10 HG embryos was reduced by 34% compared to day-10 NT embryos (*p* = 0.034). The average weight of the HG embryos was 1.35 g compared to the average weight of the NT embryos of 2.11 g. The mean body length was reduced by 14% compared to the NT group embryos (*p* = 0.041); the mean body length of the HG embryos was 35.6 mm, compared to the average for the NT embryos, 43.4 mm.

### 3.3. Morphometric and Histological Analyses of Embryonic Heart

**Figure 2:**
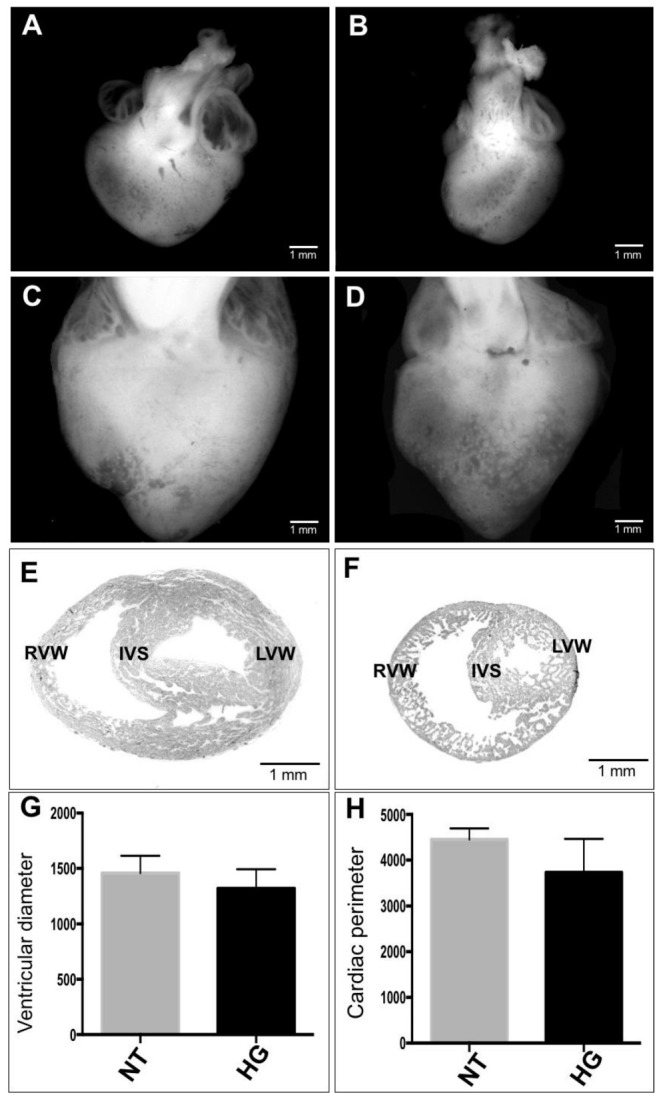
Delayed heart development in the hyperglycemic (HG) embryos compared to the control (NT) group embryos. Hearts from (**A**) a stage 32HH NT group embryo, (**B**) a stage 32HH HG group embryo, (**C**) a stage 36HH NT group embryo, and (**D**) a stage 36HH HG group embryo (all at 25× magnification). Scale bar = 1 mm. (**E**,**F**) Representative hematoxylin and eosin (HE)-stained cross-sections of (**E**) an NT embryonic heart at stage 36HH and (**F**) an HG embryonic heart at stage 36HH. Scale bar represents 1 mm. (**G**) Average ventricular diameter of the HG and NT embryonic hearts. (**H**) Average circumference of the HG and NT embryonic hearts (HG, n = 8 and NT, n = 9). Scale bar = 1 mm.

Morphometric analysis of day 10 embryos revealed that the whole heart was smaller in the HG embryos than in the NT embryos (Figure 2A–D). Cross-sections of the heart (Figure 2F,G) also revealed a slight decrease in ventricular diameter among the HG group embryos compared to the NT group embryos, in addition to a small reduction in cardiac circumference, albeit not statistically significant (Figure 2G,H).

**Figure 3:**
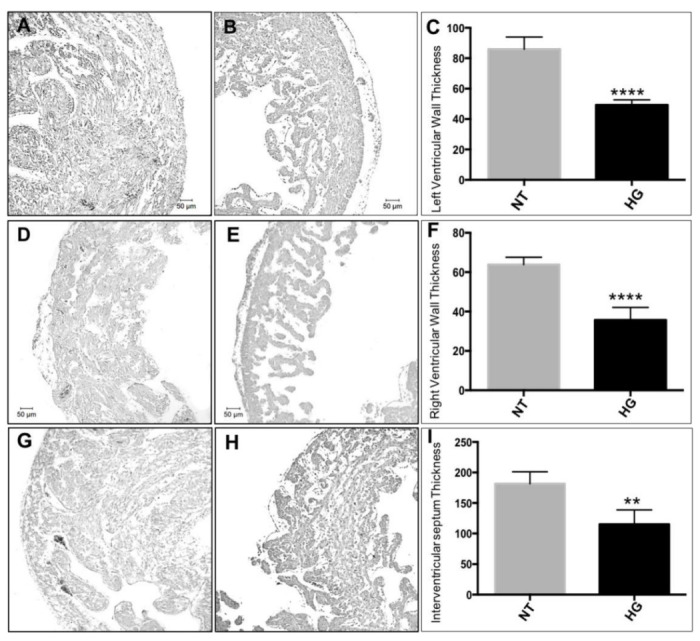
Morphometric abnormalities in hearts in typical HE-stained cross-sections of Gallus embryos incubated for 10 days. (**A**) Left ventricular wall of an NT embryo. (**B**) Left ventricular wall of an HG embryo. (**C**) Average left ventricular wall thickness of the HG and NT embryos. (**D**) Right ventricular wall of an NT embryo. (**E**) Right ventricular wall of an HG embryo. (**F**) Average right ventricular wall thickness of the HG and NT embryos. (**G**) Interventricular septum of an NT embryo. (**H**) Interventricular septum of an HG embryo. (**I**) Average interventricular septum thickness of the HG and NT embryos. All images were acquired at 40× magnification (scale bar = 200 μm). (**) *p* < 0.01, (****) *p* < 0.0001.

In addition, myocardial wall size was significantly smaller in the HG group than in the NT group at stage 36HH, with a 40% reduction in left ventricular wall thickness (*p* = 0.0002), a 35% reduction in right ventricle wall thickness (*p* = 0.0008), and a 38% reduction in interventricular septum thickness (*p* = 0.0006) (Figure 3C–I).

**Scheme 2:**
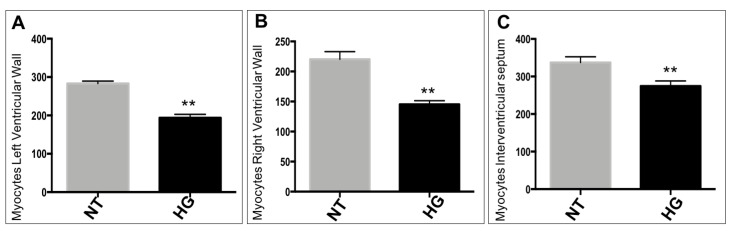
Average myocyte count per field of the HG and NT hearts (40×). (**A**) Left ventricular wall. (**B**) Right ventricular wall. (**C**) Interventricular septum. (**) *p* < 0.01.

In addition to these deficits in gross morphology, the HG group hearts also exhibited a 34% reduction in myocyte number per microscopic field across the anterior right and left ventricular walls (*p* = 0.006) and a 19% reduction in the interventricular septum (*p* = 0.004) (Scheme 2).

**Figure 4:**
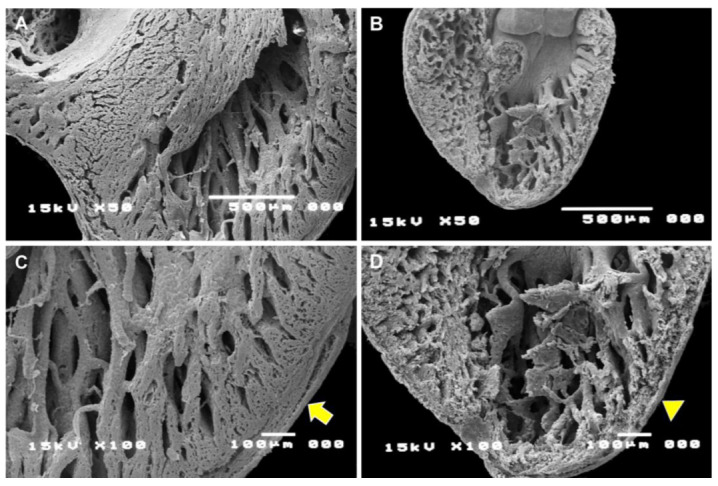
Scanning electron micrographs of longitudinal sections of the *Gallus* embryonic heart at stage 36HH showing delayed delamination and compaction of the trabeculae under hyperglycemia. (**A**) NT heart (50×), (**B**) HG heart (50×), (**C**) NT heart (100×), and (**D**) HG heart (100×). The arrow head indicates the normal thickness of the cardiac walls in NT embryos, the arrowhead indicates the decreased thickness of the ventricular walls.

Scanning electron microscopy analysis of sections from longitudinally dissected hearts revealed delayed development and thickening of the ventricular walls in the HG group (Figure 4). Furthermore, delayed delamination and compaction of the trabeculae was observed in the HG hearts compared to the NT hearts, suggesting that the delay in the formation of the structural components of the heart led to the lower level of compaction in the ventricular walls and the thinner interventricular septum, persisting the delay in the formation of the structures that form the heart.

### 3.4. Immunodetection of Myocardial Damage Biomarkers

**Figure 5:**
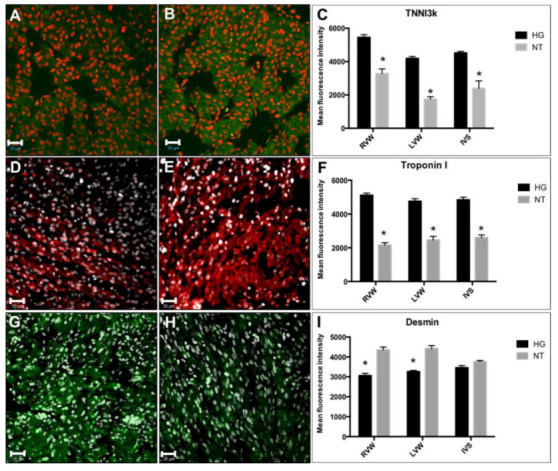
Altered expression of sarcomeric proteins in hearts of *Gallus* embryos incubated for 10 days under hyperglycemic conditions. (**A**) Representative cross-sections of the left ventricular wall immunostained for TNNI3K in an NT embryo. (**B**) Representative cross-sections of the left ventricular wall immunostained for TNNI3K in an HG embryo. (**C**) Immunofluorescence intensities of TNNI3K in the HG and NT embryos (green = TNNI3K, red = nuclear marker). (**D**) Representative cross-sections of the left ventricular wall immunostained for troponin I in an NT embryo. (**E**) Representative cross-sections of the left ventricular wall immunostained for troponin I in an HG embryo. (**F**) Immunofluorescence intensities of troponin I in the HG and NT embryos (red = troponin, white = nuclear marker). (**G**) Representative cross-sections of the left ventricular wall immunostained for desmin in an NT embryo. (**H**) Representative cross-sections of the left ventricular wall immunostained for desmin in an HG embryo. (**I**) Immunofluorescence intensity of desmin (green = desmin, white = nuclear marker). All images were acquired at 40× magnification (n = 9 HG hearts and n = 10 NT hearts). Scale bar = 20 μm, (*) *p* < 0.05.

To investigate the effect of hyperglycemia on myocardial damage markers, we compared the expression of TNNI3K, a kinase of the MAPK signaling cascade implicated in cardiomyopathies, between the NT and HG groups by immunofluorescence. Following hyperglycemia exposure, TNNI3K expression was upregulated by 30% in the right ventricular wall (*p* = 0.003), 37% in the left ventricular wall (*p* = 0.002), and 34% in the interventriucular septum (*p* = 0.002) of the HG group compared to the corresponding tissues from the NT group. Moreover, immunostaining revealed atypically dispersed sarcomeric proteins in the cytoplasm rather than the usual fibrillar form (Figure 5A,B). Similarly, the TNNI3K binding partner troponin I was upregulated by 39% in the left ventricular wall (*p* = 0.02), 24% in the right ventricular wall (*p* = 0.02) (Figure 5D–F), and 41% in the interventricular septum (*p* = 0.002) compared to corresponding tissues from NT embryos (Figure 5F). Again, this protein was not detected in the classic linear fibrillar pattern but rather in puncta throughout the HG group cardiomyocytes. In contrast, desmin expression was reduced by 23% in the left ventricular wall of the HG heart compared to the NT heart (*p* = 0.003) (Figure 5G–I).

**Figure 6:**
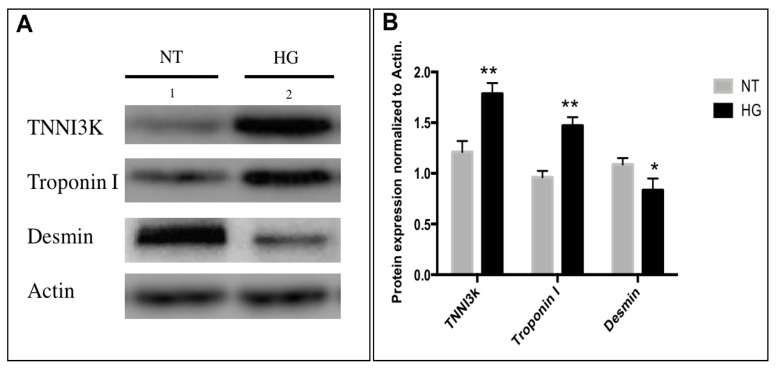
Altered expression of sarcomeric proteins in the hearts of *Gallus* embryos incubated under hyperglycemic conditions revealed by western blotting. (**A**) Representative western blots of sarcomeric proteins. (**B**) Densitometry of sarcomeric proteins. α-actin was used as the gel-loading control. (*) *p* < 0.05, (**) *p* < 0.01.

Western blot analysis of sarcomeric protein expression revealed qualitatively similar changes as TNNI3K band density increased by 62% (*p* = 0.003) and troponin density increased by 54% (*p* = 0.004) in the heart lysates from the HG group embryos compared to the NT group embryos. Additionally, the desmin band density reduced by 23% (*p* = 0.003) (Figure 6).

### 3.5. Evaluation of Cell Junction Proteins

Communication between cells is essential for cardiogenesis, the proper spatiotemporal spread of excitation and contraction, and the transmission of vascular reflex signals, among other biological functions. Cellular organization is maintained and intercellular communication is mediated by a group of junctional proteins that form occluding, adherent, and gap junctions among myocytes, and deficiencies or increases in these proteins can lead to the development of congenital cardiomyopathies, arrhythmogenesis, myocardial ischemia, arterial hypertension, and abnormal myocardial remodeling [18]. 

**Figure 7:**
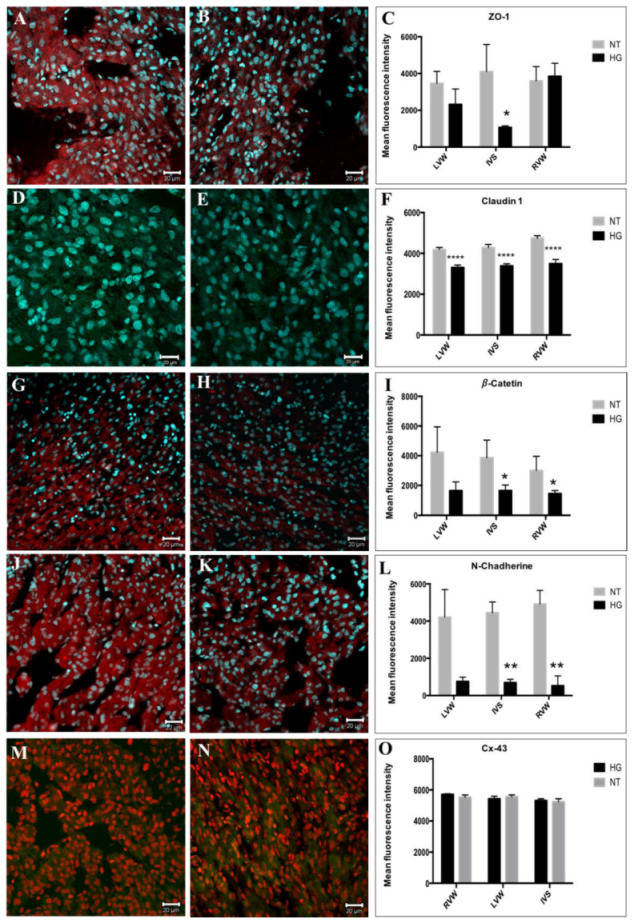
Expression levels of cell junction proteins in the heart tissue of the *Gallus* embryos. (**A**) Representative sections of the left ventricular wall immunostained for the zona occludens protein ZO-1 in an NT embryo. (**B**) Representative sections of the left ventricular wall immunostained for ZO-1 in an HG embryo. (**C**) Immunofluorescence intensity of ZO-1 in the HG and NT embryos (green = ZO-1, white = nuclear marker). (**D**) Representative sections of the left ventricular wall immunostained for the zona occludens protein claudin-1 in an NT embryo. (**E**) Representative sections of the left ventricular wall immunostained for claudin-1 in an HG embryo. (**F**) Immunofluorescence intensity of claudin 1 in the HG and NT embryos (green = claudin-1, white = nuclear marker). (**G**) Representative sections of the left ventricular wall immunostained for the zona occludens protein β-catenin in an NT embryo. (**H**) Representative sections of the left ventricular wall immunostained for β-catenin in an HG embryo. (**I**) Immunofluorescence intensity of β-catenin in the HG and NT embryos (green = B-catenin, red = nuclear marker). (**J**) Representative sections of the left ventricular wall immunostained for the zona occludens protein N-cadherin in an NT embryo. (**K**) Representative sections of the left ventricular wall immunostained for N-cadherin in an HG embryo. (**L**) Immunofluorescence intensity of N-Cadherin in the HG and NT embryos (green = N-cadherine, red = nuclear marker). (**M**) Representative sections of the left ventricular wall immunostained for the zona occludens protein Cx-43 in an NT embryo. (**N**) Representative sections of the left ventricular wall immunostained for the zona occludens protein Cx-43 in an HG embryo. (**O**) Immunofluorescence intensity of Cx-43 in the HG and NT embryos (green = Cx43, red = nuclear marker). All images were acquired at 40× magnification (n = 9 HG and n = 10 NT hearts), scale bar = 20 µm. (*) *p* < 0.05, (**) *p* < 0.01, (****) *p* < 0.0001.

To analyze changes in occluding junctions under hyperglycemia, we first measured the expression levels of ZO-1 and claudin-1 by immunostaining. The expression of ZO-1 reduced by 66% in the interventricular septum (Figure 7A–C) of the HG heart compared to the NT heart, while claudin-1 expression reduced by 22% in the RWV and 28% in the IVS of the HG heart (Figure 7D–F). Similarly, immunostaining analysis of the adherent junction protein β-catenin revealed reductions of 41% in the left ventricular wall, 38% in the interventricular septum, and 45% in the right ventricular wall of hearts from the HG embryos (Figure 7G–I), while N-cadherin immunoexpression reduced by 71% in the left ventricular wall, 74% in the interventricular septum, and 81% in the right ventricular wall (Figure 7J–L). In contrast, there were no significant differences in the expression of the gap junction protein Cx-43 between the groups, and this result was confirmed by western blotting (Figure 7M–O).

**Figure 8:**
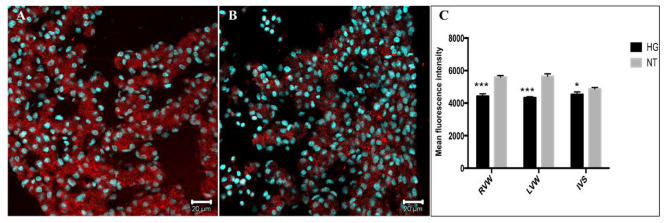
Immunostaining in transverse histological sections probed for the primary fetal glucose transporter GLUT1. (**A**) Representative image of the left ventricular wall in an NT embryo. (**B**) Representative image of the left ventricular wall in an HG embryo (both 60×; green = Glut 1 and white = nuclear marker). (**C**) Immunofluorescence intensity of GLUT1 (Scale bar = 20 µm). (*) *p* < 0.05, (***) *p* < 0.001.

We then analyzed the expression of the insulin-regulated glucose transporter GLUT1, the primary glucose transporter during embryonic and fetal development. Immunostaining revealed a 14% decrease in the left ventricular wall and an 11% decrease in the right ventricular wall of the HG embryos compared to the NT embryos, while there was no difference in expression between the HG and NT interventricular septum (Figure 8).

### 3.6. Evaluation of Cell Proliferation

**Figure 9:**
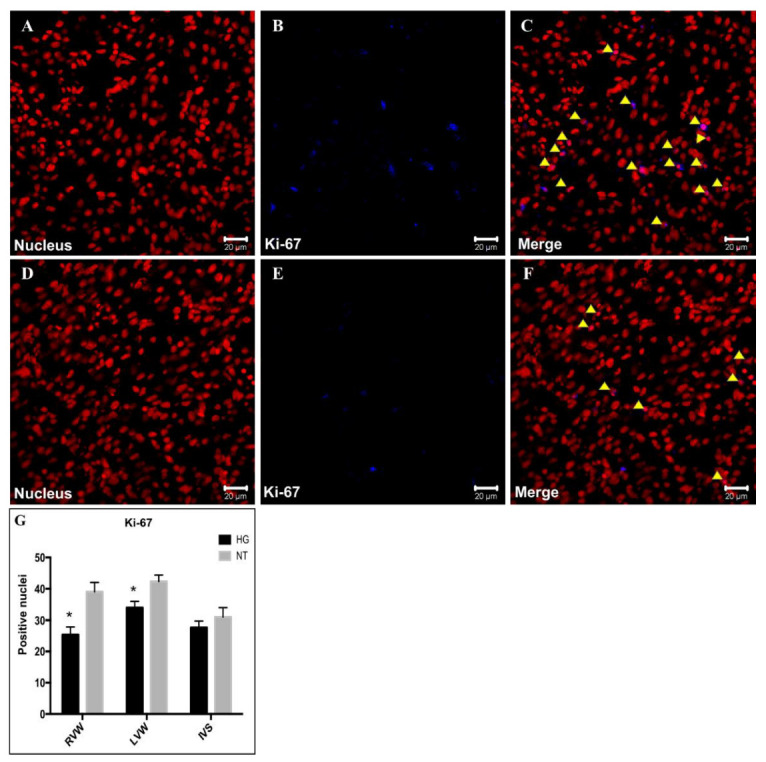
Fetal cardiomyocyte proliferation, immunostained for the proliferation marker Ki-67, in the transverse histological sections of the hearts of *Gallus* embryos incubated for 10 days. (**A**) NT embryo nuclei. (**B**) Ki-67 signal from an NT embryo. (**C**) Merge of Ki-67 positive nuclei in NT embryos. (**D**) HG embryo nuclei. (**E**) Ki-67 signal from an HG embryo. (**F**) Merge of Ki-67 positive nuclei in HG embryos. (**G**) Number of Ki-67-positive cells (photos at 40×; red = nuclear marker, blue = Ki67 and pink = merge, marked with yellow arrows) Scale bar = 20 µm, (*) *p* < 0.05.

Ki-67 is a nuclear protein associated with cell proliferation. During the interphase, Ki-67 is detected exclusively within the cell nucleus, whereas in mitosis, it mostly translocates to the surfaces of chromosomes [19]. Under normal conditions, embryonic cardiac development is characterized by high rates of cell proliferation. Given the atrophy and lower cardiomyocyte density of the HG group cardiac tissue, we examined Ki-67 expression as an index of the proliferation rate. Consistent with the observed smaller heart dimensions and lower cell density, heart tissues from the HG embryos showed a 19% reduction in Ki-67-positive nuclei within the left ventricular wall and a 16% reduction in the right ventricular wall, while there was no difference in the interventricular septum between the NT and HG groups (Figure 9).

## 4. Discussion

The number of reproductive-age diabetic women is increasing worldwide, leading to a parallel increase in the number of fetuses and delivered children with congenital heart defects [20]. Both clinical and experimental evidence suggests that elevated glucose levels during pregnancy can cause embryonic or fetal death, as well as congenital diseases leading to prenatal death [21]. Moreover, there is accumulating evidence that maternal DM can increase cardiovascular and metabolic disease risks in adulthood as a direct consequence of the hyperglycemic intrauterine environment [22]. Furthermore, a substantial proportion of these cases may be prevented by improved glucose control during pregnancy [23]. 

Taking previous investigations into account, we aimed to distinguish the effects of a hyperglycemic environment on the chicken model from those on the human heart. We first established the similarities of embryonic development under a hyperglycemic environment in our embryonic model with maternal hyperglycemia, such as delayed embryonic development, as well as fetal and neonatal cardiomyopathies, which provide new information on the risks that gestational hyperglycemia may pose to the offspring [24,25,26]. 

In this study, we found that the impairments in cardiac growth in the chicken embryo model were similar to gestational diabetes in rodent models and humans. Macrosomia was not observed because factors such as nutrition and oxygen for embryo-fetal development, as well as growth factors of maternal and placental origin, were not involved in the current model mentioned in the study.

Despite these grim statistics, the underlying molecular mechanisms remain largely unknown. Here, we present evidence that hyperglycemia during gestation induces cardiac microsomy regardless of maternal glucose status, possibly by inducing TNNI3K overexpression, as transgenic mouse studies have shown that TNNI3K overexpression reduces sarcomere length, leading to progressive cardiomyopathies and heart failure [27,28]. 

### 4.1. Importance of the Chicken Embryo Hyperglycemia Model

Experimental research on human embryos is limited by ethical considerations, while murine models do not allow for the analysis of fetal hyperglycemia independently of maternal hyperglycemia. As an alternative, the chick embryo permits precise control of fetal glucose and large-scale harvesting of heart tissue for molecular analyses. Furthermore, the results presented here are in accord with epidemiological observations in humans and experimental studies in model animals. Hyperglycemia resulted in delayed development of both the embryo as a whole and of the heart compared to the controls (Figure 1 and Scheme 1). Similarly, studies have reported that hyperglycemia in pregnant women stunts fetal growth and prenatal development, leading to microsomy [28]. In addition, recent research has suggested that in utero hyperglycemia can affect systolic and diastolic functions, leading to heart failure [29]. In rats, embryonic microsomy following hyperglycemia induced by the administration of streptozotocin on day 5 of gestation has been previously reported [30]. Therefore, the chick embryo appears to be a suitable preclinical model to study the molecular basis of congenital heart defects due to embryonic hyperglycemia.

### 4.2. Effects of Hyperglycemia on Cardiac Morphology and Histology

A delay in the morphological and functional maturation of the heart can lead to maladaptive remodeling and even cardiomyocyte hypertrophy [27,31]. The fleshy trabeculae are irregular muscular structures that attach to the ventricular wall and provide resistance to increase the force of contraction. Delay or failure of trabecular delamination and compaction is associated with contraction deficits and arrhythmias [32]. We found that heart size was reduced by hyperglycemia (Figure 2), while trabecular delamination was delayed and myocardial compaction reduced (Figure 5 and Figure 6), indicating that effects of hyperglycemia on heart morphology (Figure 2 and Figure 3) can have deleterious repercussions at the molecular and functional levels.

Proper myofibril organization is also critical for optimal myocardial contraction. Previous experimental studies have found differences in the histological characteristics of the heart at E18 among the offspring of diabetic female mice compared to controls [33]. Consistent with their findings, we found that the number of nuclei in ventricular walls markedly reduced in the HG group heart (Scheme 2) and that the number of nuclei immunopositive for the mitotic marker Ki-67 reduced substantially (Figure 9). Therefore, we conclude that hyperglycemia decreases ventricular size in part by reducing the rate of cardiomyocyte proliferation.

TNNI3K is a heart-specific MAPK kinase that regulates myocardial contraction by binding to and phosphorylating cardiac troponin I [27,34]. Excessive expression of TNNI3K is strongly implicated in the progression of cardiomyopathies [27,31]. Additionally, a previous in vivo study reported cardiomyopathy and heart failure in transgenic mice overexpressing TNNI3K [35]. Subsequently, another study reported that, in addition to cardiomyopathy, transgenic mice overexpressing TNNI3K have high plasma levels of troponin I and a greater number of heart attacks [36]. Furthermore, they showed that increased expression of TNNI3K reduced sarcomere length and promoted changes in titin composition, indicative of cardiac remodeling. They also found that TNNI3K was located in the intercalary disks of the sarcomere. In accordance with the above-mentioned reports, this study further emphasizes a relation between increased TNNI3K expression and cardiomyopathies because TNNI3K expression was significantly elevated in chicken embryos exposed to hyperglycemia (Figure 5) and exhibited abnormalities in both gross heart morphology and histological structure (Figure 3 and Figure 4). Moreover, overexpression of TNNI3K was associated with altered expression of other sarcomeric proteins, namely troponin I and desmin, as evidenced by immunofluorescence and western blotting analysis (Figure 5 and Figure 6).

### 4.3. Altered Expression Patterns of Sarcomeric Proteins Associated with Myocardial Damage

Cardiomyocytes contain linear arrays of sarcomeres aligned in parallel with the long axis of the cell, and disruption of this structural conformation by aberrant protein phosphorylation is associated with cardiac hypertrophy [37]. There is also evidence that abnormal remodeling of the postnatal heart is caused by the activation of protein kinase cascades converging on MAPKs, resulting in the phosphorylation of various cell growth and differentiation factors. The overexpression of TNNI3K is known to accelerate cardiac dysfunction in mice by inducing cardiac remodeling at the molecular level, including a reduction in sarcomere length and changes in the composition of the titin isoform [31]. Troponin I promotes actin–myosin coupling during cardiac contraction, and enhanced troponin I sensitivity has been detected following myocardial injury or infarction [38]. Troponin I and TNNI3K colocalize at the sarcomere of cardiac cells and act as effectors in the coupling of myosin and actin during contraction [31]. Overexpression of these proteins is considered an indicator of myocardial damage and a predictor of infarction [36,39]. Likewise, high troponin I expression has been reported in autopsy tissue of adult humans with diabetes and deficiency in cardiac function. Additionally, troponin I was associated with abnormal cardiac remodeling in a rat model of cardiomyopathy [40,41], which allows us to speculate that the elevated expression of troponin I, a heart-specific protein, could also be elevated in blood circulation. Based on these reports, we speculate that the elevated expression levels of TNNI3K (Figure 6A–C) and troponin I in the HG group (Figure 8) observed in this study may be indicative of myocardial damage and a consequent reduction in contractile strength. To further support this hypothesis, in this study, both TNNI3K and troponin I expression levels were increased and colocalized in a punctate pattern in hyperglycemic hearts rather than in the fibrillar arrays usually observed in healthy tissues of the NT group (Figure 5A–F).

Desmin is an integral component of intermediate filaments found in the contractile apparatus, intercalary disks, nucleus, and other cellular organelles, and underexpression has been implicated in mechanical and structural abnormalities of the cytoskeleton underlying contraction deficits, underexpression is also associated with the abnormal propagation of electrical signals between heart muscle cells [41]. We found that desmin was expressed in a diffuse pattern following hyperglycemia (Figure 5) and was undetectable in intercalary disks, in contrast to some specimens from the NT group. Furthermore, overall expression was significantly reduced in the HG group compared to the NT group (Figure 5G–I).

Emerging evidence suggests that mutations in the DES gene cause different musculoskeletal disorders and cardiomyopathies. The clinical phenotypes associated with DES mutations are heterogeneous, and some of these mutations are harmful. Clinical and experimental investigations have reported that mutations in the DES gene, such as p.A120D, a desmin variant, cause defective formation of intermediate filaments in ventricular cardiomyocytes, leading to the development of arrhythmias or cardiomyopathies [42]. 

This is also supported by studies in zebrafish embryos, where desmin knockout led to the formation of disorganized cardiac muscles, defective cardiac biomechanics, and disorders in Ca^2+^ signaling [43]. Taken together, these reports and the present study highlight the importance of low levels of desmin in our model.

Cell-to-cell communication is essential for normal cardiac embryogenesis, transmission of electrical impulses, synchronization of myocardial contractile activity, and transmission of vascular reflex signals, among other biological functions [44]. Therefore, disruption of communication among cardiomyocytes, whether due to genetic mutations or acquired conditions, can lead to cardiac pathology. Both clinical investigations and experimental studies in murine and porcine models have reported that abnormal expression levels of junctional proteins responsible for cell-to-cell communication lead to the development of cardiomyopathies, arrhythmogenesis, myocardial ischemia, arterial hypertension, and abnormal myocardial remodeling [18]. In fact, these proteins are widely considered promising therapeutic targets for the treatment of cardiomyopathies [45].

The formation of intercalated disks is incomplete during embryo-fetal development of the heart; therefore, proteins that are components of cell junctions are predominantly found in the cytoplasm. For instance, the abnormal expression of protein components forming tight junctions or occluding junctions has been associated with arrhythmias in both human patients and animal models [46]. In addition, the major occludens junctional protein ZO-1 has been shown to bind multiple DI proteins, such as connexins, catenins, and vinculins [47]. Embryos exposed to hyperglycemia demonstrated a substantial reduction in ZO-1 expression within the interventricular septum (Figure 7A–C), concomitant with the aforementioned morphological and histological abnormalities, in accord with reports that loss of ZO-1 in cardiomyocytes impairs cardiac function in mice [48]. Claudins are also essential junctional proteins, as loss is associated with abnormal morphological development, remodeling, and myocardial dysfunction [48,49]. We found that claudin-1 expression was reduced in the left ventricular wall, right ventricular wall, and interventricular septum of hearts exposed to hyperglycemia (Figure 7D–F), which may underlie the observed morphological abnormalities. Furthermore, reduced cell-to-cell coupling could predispose to greater long-term functional deficits.

Adherent junctions (AJs) are composed of proteins that form indirect associations with actin filaments and microtubules of the cytoskeleton [50]. Deficiencies or increases in the expression of these proteins can lead to the development of congenital cardiomyopathies, arrhythmogenesis, myocardial ischemia, arterial hypertension, and abnormal myocardial remodeling [51]. In the heart, AJs are responsible for mechanically docking cardiomyocytes and are closely associated with gap junction plates in intercalary disks [52]. The correct mechanical function of the heart muscle depends on the AJ component N-cadherin, which is highly expressed in both the developing and mature myocardium, where it is predominantly found in the transverse region of intercalated disks and regions of contact between neighboring myocytes [53]. In murine models, loss of N-cadherin in the embryonic myocardium resulted in embryonic lethality at midgestation, accompanied by multiple embryonic anomalies, including cardiovascular defects [54], highlighting the importance of N-cadherin for optimal heart development and function. Thus, the reduced expression levels of N-cadherin and β-catenin, another AJ protein, in the right ventricular wall, left ventricular wall, and interventricular septum (Figure 7) likely also contributed to the observed morphological abnormalities.

Finally, gap junctions are arrays of transmembrane ionic channels that allow the passage of ions for electrical coupling as well as small molecules for metabolic coupling of adjacent cardiomyocytes. These structures are formed by connexins (Cx), with isoform 43 (Cx43) the most abundant in embryonic and adult cardiac tissue [55]. Connexins also form hemichannels, allowing the exchange of ions and small low molecular weight metabolites between the cytoplasm and extracellular environment, which is crucial for communication between more distant cardiomyocytes [56]. However, we did not find significant differences in expression between hyperglycemic and normoglycemic embryos (Figure 7M–O), possibly because, during the study period, intercellular coupling via gap junctions is not fully developed.

### 4.4. Effects of Embryonic Hyperglycemia on Glucose Transport in the Heart

The mammalian heart is adapted to use multiple substrates for energy. The predominant fuel is fatty acids, followed by glucose, which accounts for around 25% of ATP production in the myocardium. In the heart, the most abundant glucose transporters are GLUT1 and GLUT4 [57]. GLUT1 is located mainly in the plasma membrane and is responsible for basal cardiac glucose uptake, while GLUT4 is present mainly in intracellular vesicles at rest but translocates to the plasma membrane in response to insulin stimulation [58]. GLUT1 is the predominant glucose transporter in embryonic and neonatal hearts, and expression is constitutive. Nonetheless, the expression level is regulated by multiple physiological and pathological stimuli. For instance, hypoxia promotes an increase in GLUT1 expression. The apoptosis rate of ventricular cardiomyocytes is elevated in adult diabetics [59], potentially due to insufficient glucose transport via GLUT1 and GLUT4. This necessitates alternative energy production by the beta oxidation of free fatty acids and results in the reduced synthesis of pyruvate [60]. Expression of GLUT1 was reduced in both walls of the hyperglycemic embryonic heart (Figure 8), which, as previously reported, could paradoxically reduce glucose availability and impede development [61]. In our model, both compaction and thickening of the ventricular walls were delayed, potentially due to reduced proliferation rate in the ventricular walls, as evidenced by Ki-67 staining (Figure 9). In the intermediate and long term, these deficits could induce malformations and functional deficits in other tissues due to insufficient oxygen supply. All the aforementioned information highlights the importance of how hyperglycemia, such as in the case of gestational diabetes, where glucose can cross the placenta freely, results in the exposure of the fetus to high levels of glucose, thus leading to multiple complications in fetal development, such as defects in the formation of the nervous and circulatory systems. These changes could therefore increase the susceptibility of offspring to develop cardiometabolic diseases later in life due to epigenetic changes during fetal development [62,63]. In addition to the above, it has been shown that lipotoxicity resulting from maternal obesity is capable of activating a series of cascades of oxidative stress and proinflammatory signals that can exacerbate cardiovascular complications induced by maternal obesity in offspring during their adult life [64]. Reasons such as these make investigations that help clarify the pathological mechanisms around the complications derived from the development under a teratogenic environment of the progeny of great importance.

## 5. Conclusions

Hyperglycemia delayed the gross development of the chicken embryonic heart and disrupted the normal organization of both cellular and molecular elements. Furthermore, hyperglycemia reduced the proliferation of cardiomyocytes. These abnormalities were associated with elevated expression levels of the sarcomeric proteins TNNI3K and troponin I, as well as with downregulation of multiple cell junction proteins, suggesting that the observed abnormalities in gross morphology arise in part from dysfunctional mechanical, electrical, and metabolic coupling among cells. In addition, the hyperglycemic heart exhibited downregulation of the main glucose transporter, GLUT1, suggesting a paradoxical deficit in glucose metabolism. These findings highlight the utility of the chicken embryo as a model for investigating the pathogenesis of congenital heart defects due to gestational diabetes, as well as the risks associated with poor glucose control during pregnancy.

## Data Availability

Not applicable.
